# From Dr. Smythe

**Published:** 1897-01

**Authors:** H. G. Smythe

**Affiliations:** Bryan, Texas


### Correspondence.

---

Bryan, Texas, December 14th, 1896.

*Editors Texas Medical Journal:*

Sirs:—In reply to the question, Who was the author of the following lines?:

"God and the doctor we alike adore,
But only when in trouble, not before,
The trouble o'er both are alike requited;
God is forgotten and the doctor slighted."

I refer you to the works of John Owen; a Welchman, born in Wales in 1560, and died 1622, and became diztinguished as a writer of Latin epigrams.

The above lines are from OWEN'S *Epigrammata.* See short sketch of John Owen (1560–1622), *Epigrammatist* in *Encyclopedia Britannica*, vol. xviii, page 88.

Yours, etc.,

H. G. SMYTHE, M. D.

---

### THE JOURNAL'S PAGE.

The Journal is appreciated everywhere. *Dorsum Rubrum* is the latest for "Red Back," a title bestowed on it by an appreciative subscriber, who, in renewing his subscription, writes: "Dr. Hadra's address, 'The Mistake of Doctors,' in the December number of the *Dorsum Rubrum*, is most enjoyable reading. Dr. ———, of this place, will send you his subscription; he says that article alone is worth many times the price of the JOURNAL."

The writers continues: "I have been looking over the files of the JOURNAL, and can freely say, when taken in comparison with similar publications, that it is an honor to its publishers, and to the State of Texas. In these days, when the medical profession is in such danger of losing caste and dignity, it is a blessing and a comfort to have a JOURNAL that will fearlessly and honestly point out the dangers, and combat them. The efforts of the JOURNAL in this direction are sincerely appreciated by all who have a just pride in their calling, and I for one commend you for your courage and zeal in the cause of rational medicine, and bid you Godspeed."

---

**Many** important articles and striking stories have been secured by *Frank Leslie's Popular Monthly* for publication during the coming year, among them an illustrated paper on the "King's Daughters and Sons," by Louise Seymour Houghton, one of the leading spirits of that order.

